# No evidence of linkage to chromosome 1q42.2–43 in 131 prostate cancer families from the ACTANE Consortium

**DOI:** 10.1054/bjoc.2000.1524

**Published:** 2000-12

**Authors:** R Singh, the ACTANE Consortium

**Affiliations:** Section of Cancer Genetics, Royal Marsden NHS Trust and Institute of Cancer Research, Downs Road, Sutton, Surrey, SM2 5PT, UK

**Keywords:** prostate cancer, linkage, chromosome 1q 42.2–43, PCaP

## Abstract

Genetic linkage studies worldwide have proposed various chromosomal localizations for prostate cancer susceptibility genes. A recent study found evidence for linkage to chromosome 1q42.2–43. The aim of our study was to attempt to confirm these findings by performing linkage analysis in 131 families with multiple prostate cancer cases selected from the ACTANE (Anglo, Canada, Texas, Australia, Norway, EU Biomed) Consortium. Parametric and non-parametric linkage (NPL) analyses were performed. Two-point LOD scores failed to show evidence of linkage at any marker (maximum two-point LOD score = 0.40 at recombination fraction θ = 0.2 with marker D1S2850). Using a multipoint heterogeneity analysis, the estimated proportion of families linked to this putative locus (α) was 0% (95% CI = 0.00–0.33). Non-parametric linkage analysis also found no evidence of linkage (maximum NPL score = –0.12, *P* = 0.55). This analysis of 131 ACTANE families does not support the presence of a locus for a prostate cancer susceptibility gene at 1q42.2–43. Although we cannot rule out the existence of such a locus, analysis indicates that less than 16% of families could be linked to this region. These findings may be a reflection of the locus heterogeneity involved in this disease indicating that there are still other major susceptibility loci to be identified. © 2000 Cancer Research Campaign http://www.bjcancer.com


*British Journal of Cancer* (2000) **83**(12), 1654–1658
© 2000 Cancer Research Campaign
doi: 10.1054/ bjoc.2000.1524, available online at http://www.idealibrary.com on IDEAL®

http://www.bjcancer.com

### No evidence of linkage to chromosome 1q42.2–43 in 131 prostate cancer families from the ACTANE Consortium

**R Singh[1] and the ACTANE Consortium**

[1]Section of Cancer Genetics, Royal Marsden NHS Trust and Institute of Cancer Research, Downs Road, Sutton, Surrey, SM2 5PT, UK; See Appendix

**Summary** Genetic linkage studies worldwide have proposed various chromosomal localizations for prostate cancer susceptibility genes. A recent study found evidence for linkage to chromosome 1q42.2–43. The aim of our study was to attempt to confirm these findings by performing linkage analysis in 131 families with multiple prostate cancer cases selected from the ACTANE (Anglo, Canada, Texas, Australia, Norway, EU Biomed) Consortium. Parametric and non-parametric linkage (NPL) analyses were performed. Two-point LOD scores failed to show evidence of linkage at any marker (maximum two-point LOD score = 0.40 at recombination fraction $\theta$ = 0.2 with marker D1S2850). Using a multipoint heterogeneity analysis, the estimated proportion of families linked to this putative locus ($\alpha$) was 0% (95% CI = 0.00–0.33). Non-parametric linkage analysis also found no evidence of linkage (maximum NPL score = – 0.12, *P* = 0.55). This analysis of 131 ACTANE families does not support the presence of a locus for a prostate cancer susceptibility gene at 1q42.2–43. Although we cannot rule out the existence of such a locus, analysis indicates that less than 16% of families could be linked to this region. These findings may be a reflection of the locus heterogeneity involved in this disease indicating that there are still other major susceptibility loci to be identified. © 2000 Cancer Research Campaign http://www.bjcancer.com

**Keywords:** prostate cancer; linkage; chromosome 1q 42.2–43; PCaP

Many case-control and cohort studies dating as far back as 1956 have observed the familial aggregation of prostate cancer. Segregation analyses (e.g. Steinberg et al, 1990) have given support for the importance of genetic factors in prostate cancer development. Various models for the mode of inheritance have been proposed. A cohort study by Monroe et al (1995) suggested that an X-linked or autosomal recessive susceptibility gene or genes may be involved. However, a complex segregation analysis performed by Carter et al (1992) found evidence for a rare highly penetrant prostate cancer susceptibility gene which is inherited in an autosomal dominant fashion. Two other studies (Gronberg et al, 1997; Schaid et al, 1998) found further support for a high-risk dominant model.

Linkage studies have found some evidence for various loci for major prostate cancer susceptibility genes. The first chromosomal localization of a putative prostate cancer gene, HPC1 (hereditary prostate cancer 1) was suggested by Smith et al (1996) from a genome wide search in 91 prostate cancer families. This study found evidence of linkage of the disease to markers on chromosome 1q24–25, with an estimated 34% of these families being linked. Although three other groups have found some confirmatory evidence for linkage in a similarly small proportion of such families (Cooney et al, 1997; Hsieh et al, 1997; Neuhausen et al, 1999), other studies have failed to find evidence for linkage at this locus (Eeles et al, 1998; Cannon-Albright and Neuhausen, 1997; McIndoe et al, 1997; Thibodeau et al, 1997). A recent meta-analysis combining these published and new data from these groups has estimated that only 6% of families worldwide are linked to this locus (Xu et al, 2000). In a recent genome screen of multiplex sibships with prostate cancer, Suarez et al (2000) found nominally significant linkage at two distal markers approximately 20cM from the putative HPC1 region.

*Received 18 February 2000*
*Revised 30 August 2000*
*Accepted 6 September 2000*

*Correspondence to:* see Appendix

More recently another prostate cancer susceptibility locus has been described by Xu et al (1998) on the X chromosome at Xq27–28. This locus was estimated to account for approximately 16% of the prostate cancer families studied. A subsequent study by Lange et al (1999) has provided further support for the existence of a prostate cancer susceptibility gene in this region in a linkage study of 153 families with at least two members affected with prostate cancer.

These findings suggest that locus heterogeneity is a feature of this disease and there may be several major susceptibility loci involved.

Berthon et al (1998) reported evidence for linkage to the region 1q42.2–43 from a study of 47 French and German multiple-case families with a maximum two-point LOD score of 2.7 at marker D1S2785. Their heterogeneity analysis estimated that the proportion of families with linkage to this locus could be as high as 50%. In a stratified analysis of nine families with early-onset prostate cancer (age less than 60 years at diagnosis), multipoint LOD and NPL scores of 3.31 and 3.32 (*P* = 0.001) respectively, were obtained with an alpha of approximately 20%. They named the putative susceptibility gene in this region PCaP (predisposing gene for cancer of the prostate). Gibbs et al (1999) also performed a linkage analysis of this region in 152 prostate cancer families. Both parametric and non-parametric analysis revealed no significant evidence of linkage. Further analysis of family subsets, stratified according to mean age of diagnosis and number of affected members, did not provide any significant evidence for linkage. In a further attempt to confirm the findings by Berthon et al, Whittemore et al (1999) conducted a linkage study in 97 families with at least three affected cases using three markers. Multipoint LOD and NPL scores failed to support linkage, even when analysis was restricted to the 14 families in their set with early-onset disease. In the genome search by Suarez et al (2000), nominally significant evidence for linkage was found at two markers in the PCaP region in a subgroup of families with late-age-at-onset prostate cancer.

The aim of our study was to attempt to confirm the findings of Berthon et al (1998) by performing linkage analysis in 131 prostate cancer families from the ACTANE Consortium using microsatellite markers in the 1q42.2–43 region.

### MATERIALS AND METHODS

### Family selection

A total of 131 families were selected from the ACTANE Consortium (excluding Canada) for this study. The criteria for selection were: three or more relatives affected with prostate cancer in a family, or a relative pair affected with the disease, one or both of whom were aged 65 years or less at diagnosis. The ACTANE consortium is a collaboration between the Cancer Research Campaign (CRC)/British Prostate Group (BPG) UK Familial Prostate Cancer Study (Anglo) and other groups worldwide with prostate cancer family sets from Canada, Texas, Australia, Norway, EU Biomed. Families were collected as follows:

#### *UK group*

All families were recruited through collaborating urologists, geneticists and oncologists via the CRC/BPG UK Familial Prostate Cancer Study. Within these families 97% of cases were clinically detected and the remaining 3% were diagnosed as a result of a prostate-specific antigen (PSA) screen. Details of family history were obtained from questionnaires completed by the index case. Cases of prostate cancer were confirmed by pathology reports, self-report, medical records or death certificates.

#### *Texan group*

Index cases were patients referred to the UTMD Anderson Cancer Centre, Houston, Texas, whose diagnoses of prostate cancer were subsequently confirmed by pathology review.

#### *Australian group*

Families were recruited from the ‘Risk factors for Prostate Cancer’ study which is a population-based case-control study conducted in Melbourne, Sydney and Perth. All probands had histopathological confirmation of prostate cancer. Reported prostate cancer cases in family members were confirmed wherever possible by matching against the National Cancer Registry and National Death Index.

#### *Norwegian group*

Families were recruited from the cancer genetics clinic at the Norwegian Radium Hospital and were included if prostate cancer affected: at least three relatives in the same lineage regardless of age of onset; or a relative pair with one aged 65 years or younger at diagnosis. Diagnosis in the prostate cancer cases was verified from the medical records. For family members reported by the cases to have prostate cancer, the diagnosis was verified by the National Cancer Registry or by the medical records when possible.

#### *EU Biomed group*

These families were collected from major urological centres across Europe. These centres have been participating in the collection of families for the analysis of high- and low-penetrance genes in prostate cancer. Only those cases in whom the diagnosis of prostate cancer could be verified from histological or medical records were included in the analysis. Breakdown of families by centre, number of affected members and age at diagnosis is shown in Table 1.

**Table 1** Family breakdown by centre, age and number of affected members

| Number of cases | UK | Texas | Australia | Norway | EU Biomed | Total |
|---|---|---|---|---|---|---|
| Two affected | 32 | 3 | 14 | 1 | 1 | 51 |
| Three or four affected | 32 | 5 | 24 | 2 | 4 | 67 |
| Five or more affected | 5 | 5 | 1 | 2 | 0 | 13 |
| Total | 69 | 13 | 39 | 5 | 5 | 131 |
| [a] Age < 65 | 30 | 3 | 22 | 3 | 2 | 60 |
| [a] Age ≥ 65 | 39 | 10 | 17 | 2 | 3 | 71 |

[a]Age figures refer to average age at diagnosis of affecteds.

Full approval for this study was obtained from the Research Ethics Committee.

### Genotype analysis

DNA for analysis was extracted from lymphocytes from blood samples provided by both affected and unaffected family members in the study. All individuals were genotyped using four polymorphic markers spanning the PCaP candidate region of 1q42.2–43 as reported by Berthon et al (1998). The dinucleotides used were D1S2850-11.2cM-D1S2785-1.2cM-D1S321-6.0cM-D1S2842. Sex-averaged distances between markers were taken from genetic maps from the Marshfield Medical Research Foundation (Broman et al 1998). Samples from all collaborating groups were genotyped at one centre.

PCR was performed as follows: one primer of each pair was radiolabelled with $^{32}$P using $^{32}$P (γ-dATP) and T4 polynucleotide kinase (PNK) in the presence of PNK-ligase buffer; the reaction mixture contained 1.5 μl of KCL Tris PE Buffer (× 10), 0.60 μl 1.0 mM $MgCl_2$ (Perkin Elmer) for markers D1S321 and D1S2785 / 2.0 mM $MgCl_2$ for marker D1S2850/ 1.5 mM $MgCl_2$ for marker D1S2842, 0.15 μl (final concentration 1.0 mM) total deoxynucleotide triphosphates (Stratagene), 0.15 μl of each primer (Oswel) at final concentration of 0.30 μM, 0.6 units Perkin Elmer gold Taq Polymerase, 5 μl genomic DNA (5 ng $\mu l^{-1}$) and water to a total volume of 15 μl. PCR was performed on a Hybaid thermocycler using annealing temperatures optimized for each primer, for a total of 35–40 cycles. PCR products were loaded onto 6% denaturing polyacrylamide gels. Controls of known size were loaded at regular intervals to act as size markers. Gels were run at 80 W for 2.5–3.0 h, dried and then exposed to X-ray film for 12 h to 3 days. Alleles were then scored visually by one observer and confirmed by a second observer. Allele scores were assigned on the basis of comparison with the CEPH family member 1347-02 who was used as a control individual. Control allele frequencies for each marker were derived from the CEPH family database (Dausset et al, 1990)

### Statistical analysis

Linkage of prostate cancer to the region 1q42.2–43 was first assessed by parametric LOD score analysis, based on the prostate-cancer-

© 2000 Cancer Research Campaign 

susceptibility model suggested by Carter et al (1992). This model assumes that prostate cancer susceptibility is due to a dominant gene with a population frequency of 0.003 and an overall penetrance of 88% by 85 years of age in carriers. This was also the basis of the models used by Berthon et al (1998) in their linkage analysis. Two-point LOD scores were calculated using Fastlink (Cottingham et al, 1993; Schäffer et al, 1994). Multipoint heterogeneity LOD (HLOD) scores were computed using Genehunter (Kruglyak et al, 1996) over the 18 cM distance spanned by the four markers. Families too large for exact computation by Genehunter were analysed using Vitesse (O'Connell et al, 1995).

Due to doubt regarding the appropriate model for age-specific prostate cancer susceptibility, non-parametric linkage scores were calculated to compare identical-by-descent sharing among all affecteds in a pedigree, with that expected under no linkage.

Analysis was performed across the whole family set and then in subsets stratified according to: number of affected men per family; and average age at diagnosis (age < 65 years and age ≥ 65 years).

### RESULTS

LOD scores from the two-point parametric analysis are given in Table 2. The results show no evidence for linkage at any marker. The largest positive LOD score was 0.40 at DIS2850 with recombination fraction θ = 0.2.

Non-parametric multipoint analysis using Genehunter found no evidence of linkage to 1q42.2–43 at marker D1S2785 either for all families combined (multipoint NPL score = –0.12, *P* = 0.55) or separately, according to number of cases affected per family: two cases (multipoint NPL score = –0.44, *P* = 0.67); three or four cases (multipoint NPL score = 0.08, *P* = 0.46); ≥ five cases (multipoint NPL score = 0.30, *P* = 0.35). Analysis was also performed in family subsets according to average age at diagnosis: under 65 years (multipoint NPL score = 0.12, *P* = 0.45) and 65 years and over (multipoint NPL score = –0.27, *P* = 0.60).

The results of the multipoint heterogeneity analyses are shown in Tables 3 and 4. The results do not provide any evidence of linkage at 1q42.2–43, the overall HLOD score for all 131 families was 0.00 and the estimated proportion linked (α) was 0 (95% CI = 0.00–0.33). For families with five or more cases, the HLOD was 0.01, with α = 0.16 (95% CI = 0–1); for families with only two cases the HLOD was also 0.00, with α = 0 (95% CI = 0.00–0.57). Analysis stratified by age at diagnosis showed that for the 60 families that had at least two members diagnosed at age 65 years or less, HLOD was 0.00 (α = 0) while the HLOD for the other 71 families was 0.01 (α = 0.07).

The results presented in Table 4 have assumed that the candidate gene lies midway between markers D1S2785 and D1S321, as was suggested by the peak LOD scores obtained in this region by Berthon's group. Therefore, we also performed heterogeneity and sub-group analyses, maximizing the position of the putative susceptibility gene over the whole 18 cM region. Results showed little change: the maximum HLOD score was only 0.37 (α = 0.32), obtained at the location of marker D1S2850, in families containing three or four cases.

**Table 2** Two-point LOD scores for four markers on chromosome 1q42.2–43

| | Recombination fraction (θ) | | | | | | |
|---|---|---|---|---|---|---|---|
| **Marker** | **0.0** | **0.01** | **0.05** | **0.1** | **0.2** | **0.3** | **0.4** |
| D1S2850 | –2.53 | –2.04 | –0.82 | –0.07 | 0.40 | 0.31 | 0.10 |
| D1S2785 | –11.39 | –9.64 | –5.94 | –3.57 | –1.31 | –0.42 | –0.08 |
| D1S321 | –0.89 | –0.65 | –0.07 | 0.26 | 0.38 | 0.24 | 0.07 |
| D1S2842 | –8.45 | –7.38 | –4.72 | –2.85 | –1.00 | –0.28 | –0.05 |

**Table 3** Multipoint linkage analysis of 131 families (non-parametric analysis)

| Marker and relative position (cM) | Multipoint Lod score | α | MultiPoint HLOD score | Multipoint NPL score | *P* |
|---|---|---|---|---|---|
| D1S2850: | | | | | |
| 0.00 | –5.31 | 0.12 | 0.05 | 0.09 | 0.46 |
| 2.25 | –4.72 | 0.11 | 0.03 | 0.05 | 0.48 |
| 4.50 | –5.01 | 0.09 | 0.02 | 0.01 | 0.49 |
| 6.75 | –5.98 | 0.07 | 0.01 | –0.03 | 0.51 |
| 9.00 | –7.86 | 0.05 | –0.00 | –0.08 | 0.53 |
| D1S2785 | | | | | |
| 11.25 | –12.41 | 0.04 | –0.00 | –0.12 | 0.54 |
| 11.50 | –12.12 | 0.04 | –0.00 | –0.12 | 0.54 |
| 11.75 | –11.91 | 0.03 | –0.01 | –0.12 | 0.55 |
| 11.99 | –11.74 | 0.03 | –0.01 | –0.13 | 0.55 |
| 12.24 | –11.62 | 0.03 | –0.01 | –0.13 | 0.55 |
| D1S321 | | | | | |
| 12.49 | –11.54 | 0.03 | –0.01 | –0.13 | 0.55 |
| 13.68 | –10.78 | 0.02 | –0.01 | –0.17 | 0.56 |
| 14.87 | –10.34 | 0.02 | –0.01 | –0.21 | 0.58 |
| 16.06 | –10.15 | 0.02 | –0.01 | –0.24 | 0.59 |
| 17.25 | –10.16 | 0.01 | –0.01 | –0.27 | 0.60 |
| D1S2842 | | | | | |
| 18.44 | –10.38 | 0.01 | –0.01 | –0.31 | 0.62 |

**Table 3** Multipoint linkage analysis of 131 families (non-parametric analysis)

| Families | Multipoint Lod score | HLOD score[a] | α | (95% CI) NPL score | *P* |
|---|---|---|---|---|---|
| All<br>0.55 | −11.91 | 0.00 | 0.00 | (0.00–0.33) | −0.12 |
| Two cases<br>0.67 | −2.33 | 0.00 | 0.00 | (0.00–0.57) | −0.44 |
| Three/four cases<br>0.46 | −8.95 | 0.01 | 0.07 | (0.00–0.43) | 0.08 |
| five-plus cases<br>0.35 | −0.62 | 0.01 | 0.16 | (0.00–1.00) | 0.30 |
| Age ≤ 65<br>0.45 | −7.07 | 0.00 | 0.00 | (0.00–0.43) | 0.12 |
| Age > 65<br>0.60 | 24.84 | 0.01 | 0.07 | (0.00–0.49) | 20.27 |

[a]Assuming heterogeneity

### DISCUSSION

Analyses of the set of 131 ACTANE prostate cancer families overall showed no significant evidence of linkage to 1q42.2–43 region using four markers flanking the putative PCaP region (two-point LOD = –0.89 to –11.39, across the region of maximum multipoint LOD reported by Berthon et al, 1998). Overall the estimated proportion of families linked was zero, although we could not confidently exclude values less than one-third. Stratification of the family set into sub-groups according to average age at

*© 2000 Cancer Research Campaign*

diagnosis and number of affected family members also yielded no evidence of linkage.

Our findings are similar to those of Gibbs et al (1999) and Whittemore et al (1999) who also failed to find evidence of linkage after analysis according to a similar stratification. Such conflicting evidence regarding linkage to this region on chromosome 1 may be influenced by various factors, particularly with respect to the sample set. Indeed, some differences exist in the profiles of families used in this study. Berthon's group used 47 families of French and German origin only while our study comprised 131 families of both European and North American origin. All French and German families studied had a minimum of three members affected with the disease, while we included families with only two members affected. However, it seems unlikely that these differences alone could account for the discrepancy in results found between the two groups. Whittemore et al (1999) used only three markers to genotype 82 families of predominantly American origin. Similarly, out of the 152 families used in the study by Gibbs et al (1999) only six were of non-white origin, the rest were white American. Thus our study used a wider cross-section of families of diverse international origin.

Berthon et al (1998) also looked for loss of heterozygosity (LOH) at 1q42.2–43 in addition to linkage. Allelic loss in this region was seen in 11 tumours, of which five also had overlapping alterations in the 1q24–25 area. This finding of LOH is of particular interest because there is another putative locus, HPC1 at 1q24–25 (Smith et al, 1996). We have yet to carry out LOH studies, but this will be undertaken as part of a future study.

In conclusion, this analysis of 131 multiple-case prostate cancer families failed to show evidence of linkage at chromosome 1q42.2–43. It is becoming increasingly evident from the results of recent linkage studies in this field that several major loci may be involved in the increased susceptibility to inherited prostate cancer. Thus, in the presence of such genetic heterogeneity, it may be difficult for different groups to replicate linkage to an infrequent locus which may account for only a small proportion of families. Evaluation of further extended pedigrees and meta-analyses of large data sets are therefore crucial in the successful identification of prostate-cancer-susceptibility loci.

### ACKNOWLEDGEMENTS

UK. We would like to thank all the patients and their families who took part in his study and all the urologists who helped with the collection of the family set. The work was supported by the Cancer Research Campaign, UK and the Prostate Cancer Charitable Trust. The genotyping and PCR machines were supported by the Times Christmas Appeal and the Prostate Cancer Charity.

Texas. We would like to thank the Department of Epidemiology who initiated the study of familial prostate cancer and the patients and their families for their participation. M Badzioch was supported by an NCI Pre-doctoral Fellowship in Cancer Prevention (R25), Dr Robert Chamberlain was the principle investigator.

Australia. We would like to thank all the men and their families who took part in this study. The work was supported by the National Health and Medical Research Council, Tattersall's and the Ted Whitten Foundation.

EU Biomed. Sample collection for the EU Biomed and analysis of the ACTANE families was supported in part by the EU Biomed programme.

### REFERENCES

Berthon P, Valeri A, Cohen-Akenine, Drelon E, Paiss T, Wohr G, Latil A, Millasseau P, Mellah I, Cohen N, Blanche H, Bellane-Chantelot C, Demenais F, Teillac P, Le Duc A, de Petriconi R, Hautmann R, Chumakov I, Bachner L, Maitland NJ, Lidereau R, Vogel W, Fournier G, Mangin P, Cussenot O, et al (1998) Predisposing gene for early-onset prostate cancer, localized on chromosome 1q42.2–43. *Am J Hum Genet* **62**: 1416–1424

Broman KW, Murray JC, Sheffield VC, White RL and Weber JL (1998) Comprehensive human genetic maps: individual and sex-specific variation in recombination. *Am J Hum Genet* **63**: 861–869

Cannon-Albright L and Neuhausen S (1997) Prostate cancer susceptibility locus on chromosome 1 examined in Utah kindreds. Paper presented at *Genetic Approaches to Breast and Prostate Cancer* Cambridge Symposia, March 22–April **17**: Cambridge

Carter BS, Beaty TH, Steinberg GD, Childs B and Walsh PC (1992) Mendelian inheritance of familial prostate cancer. *Proc Natl Acad Sci USA* **89**: 3367–3371

Cooney KA, McCarthy JD, Lange E, Huang L, Miesfeldt S, Montie JE, Osterling JE, Sandler HM and Lange K (1997) Prostate cancer susceptibility locus on chromosome 1q: a confirmatory study. *J Natl Cancer Inst* **89**: 955–959

Cottingham RW Jr, Idury RM and Schäffer AA (1993) Faster sequential genetic linkage computations. *Am J Hum Genet* **53**: 252–263

Dausset J, Cann H, Cohen D, Lathrop M, Lalouel JM and White R (1990) Centre d'etude du polymorphisme humain (CEPH): collaborative genetic mapping of the human genome. *Genomics* **6**: 575–577

Eeles RA, Durocher F, Edwards S, Teare D, Badzioch M, Hamoudi R, Gill S, Biggs P, Dearnaley D, Ardern-Jones A, Dowe A, Shearer R, McLennan DL, Norman RL, Ghadirian P, Aprikian A, Ford D, Amos C, King TM, Labrie F, Simard J, Narod SA, Easton D and Foulkes WD (1998) Linkage analysis of chromosome 1q markers in 136 prostate cancer families. *Am J Hum Genet* **62**: 653–658

Gibbs M, Chakrabarti L, Stanford JL, Goode EL, Kolb S, Schuster EF, Buckley VA, Shook M, Hood L, Jarvik GP and Ostrander EA (1999) Analysis of Chromosome 1q42.2–43 in 152 families with high risk of prostate cancer. *Am J Hum Genet* **64**: 1087–1095

Grønberg H, Damber L, Damber JE and Iselius L (1997) Segregation analysis of prostate cancer in Sweden: support for dominant inheritance. *Am J Epidemiol* **146**: 552–557

Hsieh CL, Oakley-Girvan I, Gallagher RP, Wu AH, Kolonel LN, Teh CZ, Halpern J, West DW, Paffenbarger RS Jr and Whittemore AS (1997) Re: prostate cancer susceptibility locus on chromosome 1q: a confirmatory study. *J Natl Cancer Inst* **89**: 1893–1894

Kruglyak L, Daly MJ, Reeve-Daly MP and Lander ES (1996) Parametric and nonparametric linkage analysis: a unified multipoint approach. *Am J Hum Genet* **61**: 347–353

Lange EM, Chen H, Brierley K, Perrone EE, Bock CH, Gillanders E, Ray ME and Cooney KA (1999) Linkage analysis of 153 prostate cancer families over a 30-cM region containing the putative susceptibility locus HPCX. *Clin Cancer Res* **12**: 4013–4020

McIndoe RA, Stanford JL, Gibbs M, Jarvik GP, Brandzel S, Neal CL, Li S, Gammack JT, Gay AA, Goode EL, Hood L and Ostrander EA (1997) Linkage analysis of 49 high-risk families does not support a common familial prostate cancer-susceptibility gene at 1q24–25. *Am J Hum Genet* **61**: 347–53

Monroe KR, Yu MC, Kolonel LN, Coetzee GA, Wilkens LR, Ross RK and Henderson BE (1995) Evidence of an X-linked or recessive genetic component to prostate cancer risk. *Nat Med* **1**: 99–101

Neuhausen S, Farnham J, Kort E , Tavtigian SV, Skolnick MH and Cannon-Albright LA (1999) Prostate cancer susceptibility locus *HPCl* in Utah high-risk pedigrees. *Hum Mol Genet* **8**: 2437–2442

O'Connell JR and Weeks DE (1995) The VITESSE algorithm for rapid exact multilocus linkage analysis via genotype set-recording and fuzzy inheritance. *Nat Genet* **11**: 402–408

Schaid DJ, McDonnell SK, Blute ML and Thibodeau SN (1998) Evidence for autosomal dominant inheritance of prostate cancer. *Am J Hum Genet* **62**: 1425–1438

Schäffer AA, Gupta SK, Shriram K and Cottingham RW Jr (1994) Avoiding recomputation in linkage analysis. *Hum Hered* **44**: 225–237

Smith JR, Freije D, Carpten JD, Gronberg H, Xu J, Isaacs SD, Brownstein MJ, Bova GS, Guo H, Bujnovszky P, Nusskern DR, Damber JE, Bergh A, Emanuelsson M, Kallioniemi OP, Walker-Daniels J, Bailey-Wilson JE, Beaty TH, Meyers DA, Walsh PC, Collins FS, Trent JM and Isaacs WB (1996) Major susceptibility locus for prostate cancer on chromosome 1 suggested by a genome-wide search. *Science* **274**: 1371–1374

Steinberg GD, Carter BS, Beaty TH, Childs B and Walsh PC (1990) Family history and the risk of prostate cancer. *Prostate* **17**: 337–347

*© 2000 Cancer Research Campaign* 

Suarez BK, Lin J, Burmester JK, Broman KW, Weber JL, Banerjee TK, Goddard KA, Witte JS, Elston RC and Catalona WJ (2000) A genome screen of multiplex sibships with prostate cancer. *Am J Hum Genet* **66**: 933–944

Thibodeau SN, Wang Z, Tester DJ, French AJ, Schroeder JJ, Bissonet AS, Roberts SG (1997) Linkage analysis at the HPC1 locus in hereditary prostate cancer families. *Am J Hum Genet* (Suppl) **61**: A297

Whittemore AS, Lin IG, Oakley-Girvan I, Gallagher RP, Halpern J, Kolonel LN, Wu AH and Hsieh CL (1999) No evidence of linkage for chromosome 1q42.2–43 in prostate cancer. *Am J Hum Genet* **65**: 254–256

Xu JF, Meyers D, Freije D, Isaacs S, Wiley K, Nusskern D, Ewing C, Wilkens E, Bujnovszky P, Bova GS, Walsh P, Isaacs W, Schleutker J, Matikainen M, Tammela T, Visakorpi T, Kallioniemi OP, Berry R, Schaid D, French A, McDonnell S, Schroeder J, Blute M, Thibodeau S and Trent J (1998) Evidence for a prostate cancer susceptibility locus on the X chromosome. *Nat Genet* **20**: 175–179

Xu J,International Consortium for Prostate Cancer Genetics (2000). Combined analysis of hereditary prostate cancer linkage to 1q24–25: results from 772 hereditary prostate cancer families from the International Consortium for Prostate Cancer Genetics. *Am J Hum Genet* **66**: 945–957

### APPENDIX

### *ACTANE Consortium
### *All authors from ACTANE Consortium are at equal position

*The Cancer Research Campaign/British Prostate Group UK Familial Prostate Cancer Study*
R Singh[1*], SM Edwards[2*], D Teare[3], DP Dearnaley[2,4], A Ardern-Jones[4], A Murkin[4], RJ Shearer[4], Z Kote-Jarai[2], RA Jackson[2], D Easton[3 †], The CRC/BPG UK Familial Prostate Cancer Study Collaborators[**], RA Eeles[1,2 †]

[1]Section of Cancer Genetics, Royal Marsden NHS Trust and Institute of Cancer Research, Downs Road, Sutton, Surrey SM2 5PT; [2]Institute of Cancer Research, 15 Cotswold Road, Sutton, Surrey SM2 5NG; [3]CRC Genetic Epidemiology Unit, Strangeways Research Laboratories, Worts Causeway, Cambridge CB1 8RN; [4]The Royal Marsden NHS Trust, Downs Road, Sutton, Surrey SM2 5PT, UK.

*Texas*
M Badzioch[1 †], C Amos[2*]
[1]Division of Medical Genetics, University of Washington Medical Center, Box 357720, Seattle WA 98195–7720; [2]MD Anderson Cancer Center, Texas Medical Center, 1515 Holcombe Boulevard, Houston, Texas 77030, USA.

*Australia*
GG Giles[1 †], JL Hopper[2*]
[1]Cancer Epidemiology Centre, Anti-Cancer Council of Victoria, 100 Drummond Street, Carlton South 3053; [2]University of Melbourne, Cancer Epidemiology Centre, 200 Berkeley Street, Carlton, Victoria 3053, Australia.

*Norway*
K.Heimdal[†], P.Moller[†] – Unit of Medical Genetics, Norwegian Radium Hospital, Oslo N0310, Norway.

*Eu Biomed*
DT Bishop[1 †], EU Biomed Prostate Cancer Linkage Consortium[**]
[1]ICRF Genetic Epidemiology Laboratory, Ashley Wing, St James' University Hospital, Beckett Street, Leeds LS9 7TF, UK.
[†;]Principal Investigators; [*]Major Authors; [**]Lists available on request.
*Correspondence to*: RA Eeles, Institute of Cancer Research and Royal Marsden Hospital NHS Trust, 15 Cotswold Road, Sutton, Surrey, SM2 5NG, UK. Tel: +44181 661 3642; Fax: +44181 770 1489; E-mail: ros@icr.ac.uk

 *© 2000 Cancer Research Campaign*
